# Moderate Scale of Pig Farming: Implications for Herd Management and Disease Control—Evidence from Heilongjiang Province, China

**DOI:** 10.3390/vetsci13080728

**Published:** 2026-07-24

**Authors:** Chang Liu, Zengyong Zhu

**Affiliations:** Institute of Animal Science, Chinese Academy of Agricultural Sciences, No. 2 Yuanmingyuan West Road, Haidian District, Beijing 100193, China; 82101232524@caas.cn

**Keywords:** pig farming, moderate operating scale, herd management, disease control, production efficiency, environmental management

## Abstract

Moderate-scale farming is an important pathway for promoting the high-quality development of China’s pig industry. Based on survey data from 809 pig farms in Heilongjiang Province in 2024, this study assesses the moderate operating scale of pig farming within the framework of herd management and disease control, focusing on production efficiency, disease prevention and control, and environmental management. Data Envelopment Analysis (DEA) is used to measure production efficiency, and baseline regression models with operating-scale group dummy variables are constructed to compare operational performance across annual slaughter-volume groups. Compared with the other operating-scale groups, relatively better production efficiency was observed in the 5000–10,000 head group. The 3000–4999 head group shows relatively better disease prevention and control performance, with lower pig mortality, and a greater advantage in controlling manure treatment costs. Overall, the relatively better-performing scale range differs across evaluation dimensions. However, the 3000–4999 head group shows relatively stronger coordination among production organization, disease prevention and control, and environmental management, and may therefore represent a relatively suitable operating-scale range for supporting herd management and disease control in pig farming.

## Simple Summary

Defining the appropriate scale of pig farming is an important question that urgently needs to be addressed in promoting the high-quality development of China’s pig industry. Based on survey data from 809 pig farms in Heilongjiang Province, this study examines, within the framework of herd management and disease control, which annual slaughter-volume range performs relatively better when production efficiency, disease prevention and control, and environmental management are considered together. The results show that the relatively better-performing group differs across these three aspects. Farms with an annual slaughter volume of 5000–10,000 head show relatively better production efficiency, while farms with an annual slaughter volume of 3000–4999 head show relatively better disease prevention and control performance and a greater advantage in controlling manure treatment costs. Overall, the 3000–4999 head group shows stronger coordination across production organization, disease control, and environmental management, and may therefore represent a relatively suitable scale for pig farming in Heilongjiang Province at the current stage.

## 1. Introduction

The pig industry is a pillar of China’s animal husbandry sector. It plays an important strategic role in ensuring the security of livestock product supply. Under price fluctuations, environmental constraints, and epidemic shocks, pig farming in China faces considerable risks. Cyclical fluctuations have also become a salient feature of the pig industry [1]. Large-scale pig farming is an important approach to reducing fluctuations and promoting the high-quality development of China’s pig industry [2]. In this study, large-scale pig farms refer to pig farms with an annual slaughter volume of 500 head or more. However, given China’s resource endowments, an ultra-large-scale model of pig farming may not align with actual production conditions. In contrast, moderate-scale operation is more consistent with the current development needs of the pig industry. The Outline of the Fifteenth Five-Year Plan for National Economic and Social Development of the People’s Republic of China, released in March 2026, proposed developing moderate-scale agricultural operations and improving the development quality of new agricultural business entities. It also called for strengthening the comprehensive regulation of pig production capacity. This indicates that moderate-scale operation has become an important issue in promoting the high-quality development of the pig industry.

A moderate operating scale generally refers to the range at which pig farms can better coordinate economic returns, resource-use efficiency, environmental management, and disease-risk control under given technical and socio-economic conditions [3]. From a macro perspective, moderate-scale operation should be guided by resource endowments, ecological and environmental protection, animal health risks, and the stability of industrial operation. Pig farming is not merely an internal production activity. Pollution generated by farming has clear spillover effects, and without effective management, the private costs of pig farming may diverge from its public costs, thereby increasing ecological and environmental pressure and constraining the green transformation of the industry [4,5]. At the same time, the severe shock caused by the introduction of African swine fever into China’s pig industry further demonstrated that disease prevention and control are closely related not only to pork supply security and industrial stability, but also to herd health management and epidemiological risk control [6,7]. At the farm level, pig farms are rational business entities, and improving production efficiency and farming returns remains an important driver of moderate-scale operation. However, operating scale also affects the conditions under which animal health and disease prevention are managed. Changes in farm scale may influence stocking density, pig movement, personnel and vehicle flows, manure handling, cleaning and disinfection routines, and daily health monitoring [8,9]. These factors are closely related to the probability of pathogen introduction, the effectiveness of biosecurity implementation, and the timeliness of veterinary intervention [10]. Therefore, assessing the moderate operating scale of pig farming requires a balance among production efficiency, disease prevention and control, and environmental management, while also considering animal health outcomes, epidemiological risk exposure, and herd-level disease prevention capacity.

Heilongjiang Province is one of China’s major grain-producing regions and has strong potential for pig production. In 2025, its annual pig slaughter volume reached 24.406 million head, an increase of 4.0% over the previous year. However, the industry still faces several constraints, including the need to ensure stable supply, market price fluctuations, tight feed raw material supply, growing environmental pressure, and animal disease prevention and control [11]. Based on survey data from 809 specialized fattening pig farms in Heilongjiang Province, this study examines the relationship between operating scale and farm performance across three dimensions: production efficiency, disease prevention and control, and environmental management. The specific objectives are to compare the performance of different annual slaughter-volume groups in terms of production efficiency, disease prevention and control, and environmental management; to examine whether the relatively better-performing scale range differs across these dimensions; and to provide a comprehensive assessment of operating-scale performance based on the three dimensions.

The contributions of this study can be understood in both academic and practical terms. From an academic perspective, this study extends the assessment of pig-farm operating scale beyond a single economic perspective by jointly considering production efficiency, disease prevention and control, and environmental management. It provides farm-level empirical evidence that the relatively better-performing scale range may differ across evaluation dimensions and contributes to a more comprehensive understanding of operating-scale performance under multiple production and management objectives. Building on this multidimensional evidence, the study also has practical relevance. The findings provide empirical evidence for understanding the operating-scale structure of pig farming in Heilongjiang Province and may support more differentiated farm-management and policy decisions concerning production organization, herd health management, environmental management, and disease-risk control. Taken together, these contributions highlight the value of evaluating pig-farm operating scale through a multidimensional framework that considers both production performance and broader management requirements.

## 2. Materials and Methods

### 2.1. Materials

The selection of the three evaluation dimensions and the design of the subsequent survey and empirical analysis were informed by the existing literature on pig-farm operating scale. The following review summarizes the theoretical mechanisms and empirical evidence concerning the relationships between operating scale and production efficiency, disease prevention and control, and environmental management.

#### 2.1.1. The Relationship Between Operating Scale and Production Efficiency

Production efficiency measures how effectively a production unit utilizes resources to meet specific economic objectives. It represents the ratio between actual inputs and the theoretical minimum required to maintain a specific output level under given technical conditions. Consequently, production efficiency serves as an important metric for assessing the moderate operating scale of pig farming. At present, a substantial body of research has examined pig farming scale from the perspective of production efficiency. Scholars generally agree that larger operating scale is not always better and that scale expansion has certain limits [12].

As operating scale expands, pig farms can spread fixed facility investment over a larger output volume. Production processes may also become more specialized and standardized, and scale advantages in input procurement may gradually emerge. These mechanisms may improve production efficiency to some extent [13,14]. At the same time, however, expanding operating scale may also increase management costs and reduce production flexibility. Larger pig farms usually involve more management layers, higher coordination and communication costs, and a greater risk of information asymmetry. These factors may gradually weaken the efficiency gains from scale expansion [15,16].

The operating scale that maximizes production efficiency varies across regions, development stages, and farming conditions [17,18]. Based on a survey of 301 pig farmers in Hubei and Jiangxi Provinces, Pan et al. [19] found an inverted U-shaped relationship between total factor productivity and operating scale. They identified an annual slaughter volume of 1680 head as the efficiency-optimal operating scale. Based on a 2014 survey of 800 pig farms in Jiangsu Province, Yao et al. [20] found that an annual slaughter volume of 300–500 head represented an appropriate operating scale. Yan et al. [21] estimated pig production efficiency across different operating-scale groups in Eastern, Central, and Western China. Their results suggested that backyard farming and farming at larger operating scales were more suitable in western China, whereas farming at larger operating scales was more suitable in eastern and central China.

Production efficiency is an important perspective for assessing the moderate operating scale of pig farming because it reflects how effectively farms organize production and allocate resources under different scale conditions. Previous studies have provided both theoretical explanations and empirical evidence showing that the efficiency effects of scale expansion are not uniform, but vary across production conditions and regions. These findings provide an important empirical basis for collecting detailed information on production inputs and outputs and for incorporating production efficiency as one of the evaluation dimensions in this study.

#### 2.1.2. The Relationship Between Operating Scale and Disease Prevention and Control

The performance of disease prevention and control affects not only the production stability of pig farms but also the effective supply of pork and the stable operation of the pig industry [22]. As a result, disease prevention and control constitutes an important safety dimension for assessing moderate operating scale.

As rational economic agents, pig farms seek to maximize returns by allocating resources efficiently. In terms of risk awareness, expanding operating scale may increase potential losses in the event of a disease outbreak. Therefore, larger pig farms generally have stronger incentives to establish well-developed disease prevention systems, standardize biosecurity management, and reduce the probability of disease outbreaks [23]. In terms of objective conditions, larger-scale pig farms typically have stronger financial capacity, more comprehensive biosecurity measures, and more standardized prevention and control systems. These conditions may enhance their disease prevention and control capacity [24,25]. Schembri et al. found that small-scale pig farms typically adopted relatively extensive management practices and had lower levels of biosecurity [26]. Zhang et al. [27] found that, compared with free-range farmers, large-scale pig farms were generally more proactive in establishing biosecurity systems and tended to adopt a broader range of biosecurity measures.

However, expanding operating scale does not always lead to continuous improvement in disease prevention and control performance. It may also be associated with higher stocking density and a stronger concentration of disease risk. Once prevention and control measures fail, disease outbreaks may cause greater losses [28]. Li et al. [29] found that, during the ASF epidemic, small-scale and medium-scale pig farms may have had certain advantages in disease control because their operating scale was relatively limited and personnel movement was easier to manage. Yu et al. [30] found that, during avian influenza outbreaks, larger poultry farms experienced greater declines in flock size.

Overall, assessing operating scale from the perspective of disease prevention and control is necessary because herd health outcomes depend not only on the availability of preventive resources, but also on whether biosecurity measures can be implemented effectively under different production scales. The existing literature indicates that larger farms may benefit from stronger investment capacity and more standardized disease-prevention systems, while greater stocking density and more complex personnel and animal movements may simultaneously increase epidemiological pressure. This evidence guided the inclusion of pig mortality, disease-prevention investment, cleaning and disinfection frequency, biosecurity level, and surrounding disease risk in the questionnaire and subsequent empirical analysis.

#### 2.1.3. The Relationship Between Operating Scale and Environmental Management

Environmental issues have received increasing attention in China. Under increasingly stringent environmental protection requirements and the gradual internalization of pollution control costs, environmental management has become a crucial dimension in assessing the moderate operating scale of pig farming [31]. Environmental management is also closely related to the farming environment, herd health, and public health. If manure and wastewater generated during pig production are not properly collected, stored, and treated, they may not only increase the risks of nitrogen and phosphorus emissions, odor diffusion, and water pollution, but also create more opportunities for pathogenic microorganisms to persist and spread in the farm environment. This may further affect farm environmental hygiene, biosecurity implementation, and the maintenance of herd health [32,33].

Existing studies offer two main perspectives on the relationship between operating scale and environmental management. One view is that expanding operating scale can help reduce manure treatment costs, lower the risk of environmental pollution, and improve environmental quality [34,35]. The other view is that, as operating scale expands and production becomes more concentrated, the total amount of manure discharge increases and its spatial concentration becomes more pronounced. This may increase the pressure on on-farm hygiene management, manure collection and transport, and the coordinated management of biosecurity. If crop–livestock integration conditions are insufficient or the subsequent utilization chain is incomplete, the pollution load may exceed the carrying capacity of the surrounding environment and cause damage to local environmental conditions [36,37].

Scholars have reached different conclusions when assessing the moderate operating scale from the perspective of environmental management. Despite these differences, their findings generally suggest that the internalization of environmental costs narrows the range of moderate operating scale. McAuliffe et al. [38] found that when environmental performance is incorporated into the evaluation, the moderate operating scale determined solely by economic returns may change. Therefore, scale expansion should not be assessed from a single dimension. Wu et al. [39] found that, without considering environmental pollution control costs, the optimal operating scale in Funing County, Jiangsu Province, was an annual slaughter volume of 600–800 head. However, after these costs were internalized, the optimal operating scale decreased to 31–35 head. From the perspective of minimizing environmental pollution, Xu et al. [40] suggested that two annual slaughter-volume ranges—less than 499 head and 1000–1999 head—could represent moderate operating scales for pig farming.

Against this background, the environmental implications of scale expansion provide another essential basis for judging whether an operating scale is moderate. Although larger farms may achieve lower unit treatment costs through specialized facilities and improved resource utilization, the concentration of manure production can also place greater pressure on collection, treatment, transport, and land application. Drawing on these findings, the survey collected information on manure treatment costs, treatment-facility investment, supporting cropland, and different forms of manure resource utilization.

Taken together, the three dimensions provide complementary perspectives for assessing the moderate operating scale of pig farming. Production efficiency reflects the organizational and resource-allocation basis of farm operation; disease prevention and control performance reflects herd health outcomes and the capacity to manage disease risks; and environmental management, particularly manure management, reflects the ability of farms to manage environmental hygiene, treatment costs, and associated biosecurity risks.

Despite the insights provided by the existing literature, several research gaps remain. First, existing studies have often examined pig-farm operating scale from a single perspective, while relatively limited attention has been paid to integrated assessments within the framework of herd management and disease control. Under actual production conditions, moderate operating scale concerns not only economic performance, but also the capacity of pig farms to implement disease prevention and control measures and organize environmental management effectively. Second, empirical evidence from Heilongjiang Province remains limited, and the applicability of findings from other regions requires further examination.

Therefore, based on survey data from 809 specialized fattening pig farms in Heilongjiang Province, this study assesses the moderate operating scale of pig farming by comparing operating-scale performance across three dimensions: production efficiency, disease prevention and control, and environmental management, within the framework of herd management and disease control.

### 2.2. Survey

#### 2.2.1. Study Area

Heilongjiang Province is located in northeastern China, approximately between 121°11′–135°05′ E and 43°26′–53°33′ N. As one of China’s major grain-producing regions, Heilongjiang has a strong foundation for grain production and a relatively sufficient supply of feed crops. These conditions provide favorable resource endowments for the development of pig farming.

**Figure 1:**
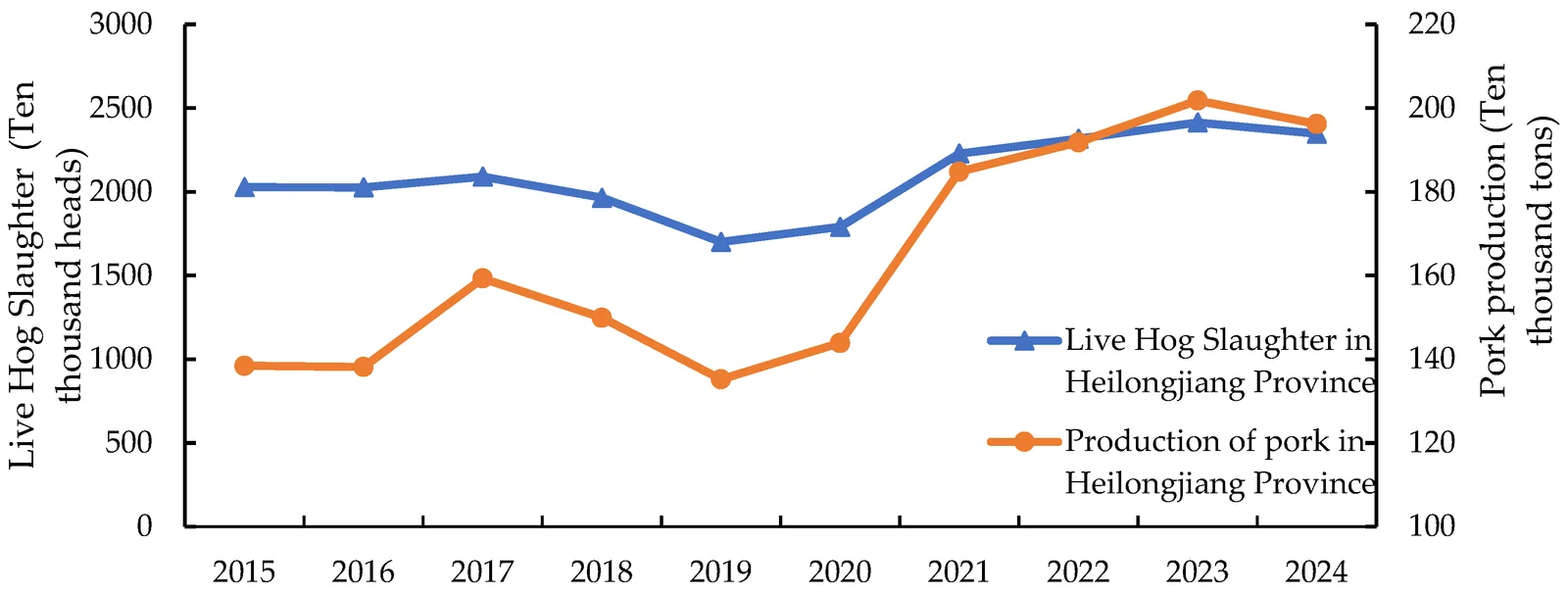
Live hog slaughter and pork output in Heilongjiang Province, 2015–2024. (Data source: obtained from the National Data database of the National Bureau of Statistics of China [41]).

**Figure 2:**
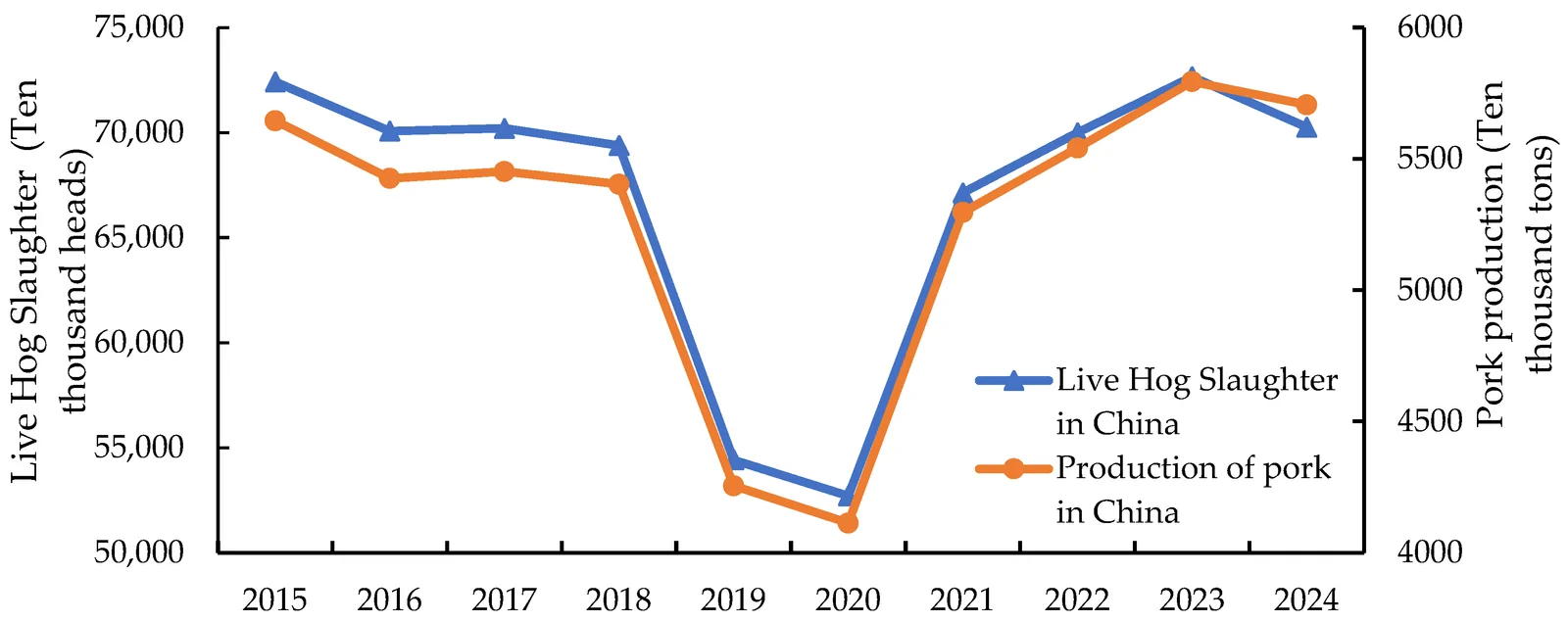
Live hog slaughter and pork output in China, 2015–2024. (Data source: obtained from the National Data database of the National Bureau of Statistics of China [41]).

**Figure 3:**
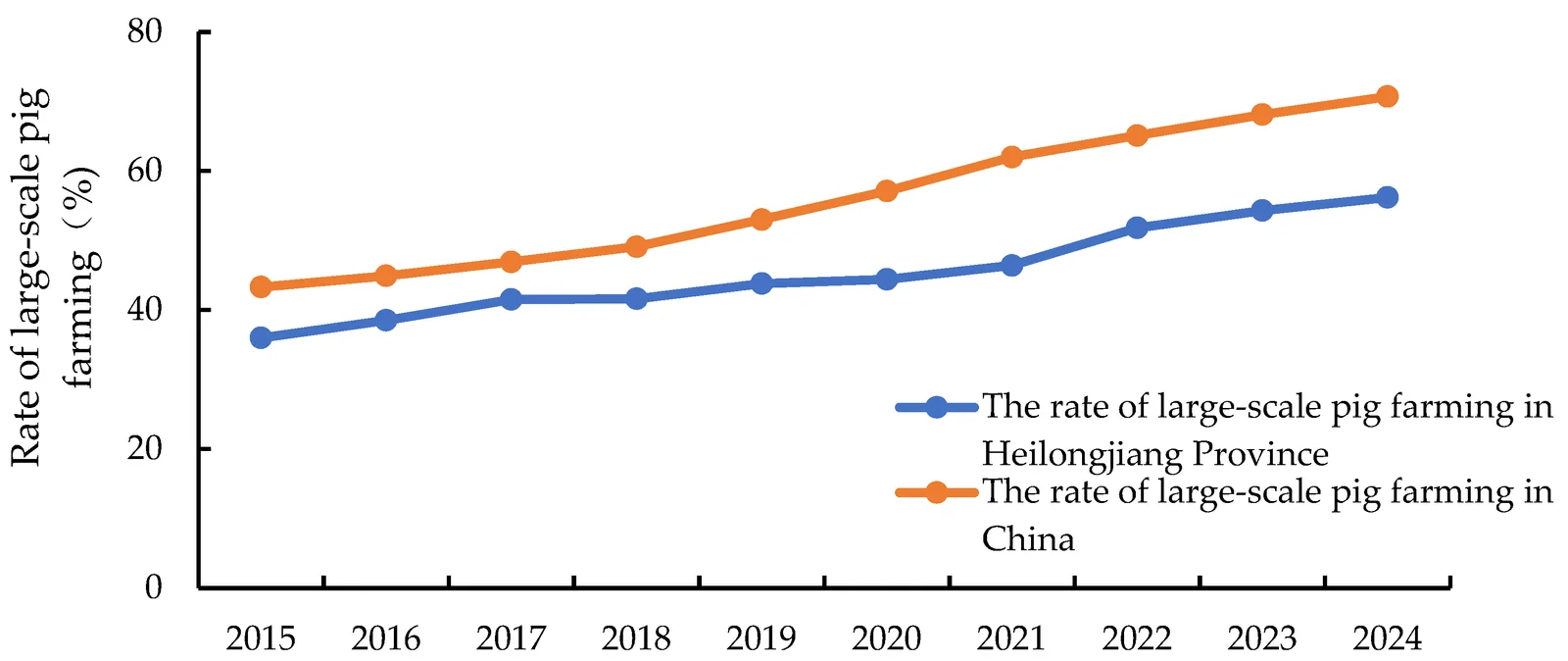
Share of large-scale pig farming in China and Heilongjiang Province, 2015–2024. (Data source: compiled from the relevant annual editions of the China Animal Husbandry and Veterinary Yearbook [42]).

In recent years, pig farming in Heilongjiang has developed rapidly, although the share of large-scale pig farming remains lower than the national average. In terms of annual slaughter volume and pork output, the number of pigs slaughtered in Heilongjiang increased from 20.276 million in 2015 to 23.467 million in 2024, with an average annual growth rate of 1.6% (Figure 1). Pork output also increased from 1.384 million tons to 1.962 million tons during the same period. These growth rates were higher than the national averages of −0.3% and 0.1%, respectively (Figure 2). However, from the perspective of large-scale pig farming, Heilongjiang still has considerable room for improvement. The share of pig slaughter volume contributed by farms with an annual slaughter volume of 500 head or more increased from 36.0% in 2015 to 56.2% in 2024 (Figure 3), with an average annual increase of only 2.2 percentage points. By comparison, the corresponding national share increased from 43.35% to 70.7% over the same period, with an average annual increase of 3.0 percentage points. These characteristics make Heilongjiang a relevant study area for examining moderate operating scale and comparing farm performance across different operating-scale groups. All data reported in this paragraph and presented in Figure 1, Figure 2 and Figure 3 were obtained from the National Data database of the National Bureau of Statistics of China [41] and the China Animal Husbandry and Veterinary Yearbook [42].

#### 2.2.2. Questionnaire Design and Survey Implementation

The survey was conducted from July to August 2025 in Heilongjiang Province, China. This study adopted an observational, cross-sectional survey design, with the pig farm serving as the analytical unit rather than the individual pig or production batch. The target respondents were specialized fattening pig farms, which mainly purchase piglets and organize production around the fattening stage rather than operating a complete farrow-to-finish production system. In this study, annual slaughter volume refers exclusively to the number of fattening pigs marketed for slaughter by each farm during 2024. It does not include piglets, replacement gilts, breeding sows, boars, or culled breeding pigs. Farms without fattening-pig production or slaughter, including breeding farms and farms specializing only in piglet production, were not included in the survey sample. Specialized fattening farms were selected because their production inputs, cost structures, and operational boundaries are relatively clear, which facilitates comparisons of production efficiency, disease prevention and control performance, and manure treatment costs across operating-scale groups.

The questionnaire was developed with reference to the literature reviewed above and was organized around farm owner characteristics, farm characteristics, production inputs and outputs, disease prevention and control, biosecurity management, manure collection and treatment, and external production conditions. The production module collected information on slaughter volume, slaughter weight, selling price, feed cost, piglet cost, labor cost, medical and disease-prevention cost, and other production costs. The disease-prevention module covered pig mortality, disease-prevention investment, cleaning and disinfection frequency, biosecurity conditions, and surrounding disease risk. The environmental management module collected information on manure collection methods, treatment facilities, treatment costs, supporting cropland, manure resource utilization, and related subsidies.

Data were collected through field visits and face-to-face interviews. The survey focused on the production and operational conditions of the sampled farms in 2024. During each interview, trained investigators explained the questions to respondents when necessary and checked the completed questionnaire for missing information or logically inconsistent responses. The English version of the questionnaire used in the survey is provided as Supplementary Material File S1.

#### 2.2.3. Sample Classification and Variable Description

Questionnaires lacking the information required for the empirical analysis or containing responses that remained logically inconsistent after verification were excluded during data screening. After questionnaire screening and data verification, 809 valid questionnaires were retained, corresponding to a valid response rate of 86.0%.

According to the classification criteria in the *China Animal Husbandry and Veterinary Yearbook* [42], pig farms with an annual slaughter volume of 500 head or more are classified as large-scale farms under the Chinese statistical standard. Based on this criterion, and taking into account the distribution of annual slaughter volume in the sample and the grouping approaches adopted in previous studies [40], the sampled pig farms were further classified into five annual slaughter-volume groups. The 1–499 head group included 435 farms; the 500–1499 head group included 177 farms; the 1500–2999 head group included 78 farms; the 3000–4999 head group included 75 farms; and the 5000–10,000 head group included 44 farms.

The variables used in the empirical analysis cover operating scale, production inputs and outputs, disease prevention and control performance, environmental management cost, farm owner characteristics, farm characteristics, and external environmental conditions. Detailed variable definitions and descriptive statistics are provided in Supplementary Tables S1 and S2. Categorical variables are reported as counts and percentages of pig farms in each category, *n* (%), in Table S1, while continuous variables are reported as means and standard deviations (SD) in Table S2. Concise variable codes are used in the subsequent regression tables to improve readability. These codes do not alter the definitions or substantive meanings of the corresponding variables.

### 2.3. Methods

#### 2.3.1. Moderate Operating Scale from the Perspective of Production Efficiency

At present, production efficiency is mainly measured using parametric stochastic frontier analysis (SFA) and nonparametric data envelopment analysis (DEA). Compared with SFA, DEA does not require the prior specification of a production function and is more flexible in handling multiple input and output indicators. It is therefore especially suitable for efficiency evaluation involving multiple inputs and outputs. DEA also constructs the efficient frontier directly from sample data and can intuitively reflect relative efficiency differences among decision-making units. For this reason, it has been widely used in studies of agricultural operational efficiency and pig farming efficiency [18,43,44]. Given that the sample in this study consists of cross-sectional data from pig farms with different operating scales, and that the input and output indicators are multidimensional, DEA is appropriate for measuring production efficiency. The specific model is shown in Equation (1).

For the output indicator, studies on pig production generally use either pig weight per head or output value per pig. This study uses output value per pig as the output indicator, which is calculated as slaughter weight multiplied by the selling price per kilogram. The selling price used here is the actual sale price reported by each surveyed pig farm based on its own production records, rather than a regional average market price. This choice is based on two considerations. First, slaughter weight can reflect the production management and feeding organization of pig farms. Second, the selling price can reflect the market response capacity and actual sales performance of pig farms with different operating scales. Taken together, these two factors allow the production efficiency indicator to capture, as far as possible, differences in production management and sales performance across operating-scale groups.

For the input indicators, drawing on the existing literature [18,45], this study selects five major cost items: feed cost, piglet cost, labor cost, medical and disease prevention cost, and other costs. These indicators are converted into unit-based measures, so that the efficiency evaluation focuses on output performance under unit input constraints and improves comparability across operating-scale groups.

(1) $$\left[\begin{array}{l} \min\;\theta \;\\ s.t.\;\sum_{j=1}^{n}\lambda_{j}x_{ij}\leq \theta x_{i0},\;i=1,2,\cdots ,m;\;\\ \sum_{j=1}^{n}\lambda_{j}y_{j}\geq y_{0};\;\\ \lambda_{j}\geq 0,\;j=1,2,\cdots ,n \end{array}\right.$$

In Equation (1), θ represents the efficiency value of pig farms; *X_ij_* denotes the *i*-th input of the *j*-th farm; *Y_j_* represents the output of the *j*-th farm. When θ = 1, the pig farm is located on the production frontier and is DEA-efficient. When θ < 1, the pig farm deviates from the production frontier and is DEA-inefficient, indicating that there is room for input reduction under the given output level.

In the next step, θ is used as the dependent variable. Referring to Zhang et al. [46], this study constructs a multiple linear regression model with operating-scale group dummy variables. The 1–499 head group is used as the reference group to compare conditional differences in production efficiency across other operating-scale groups. The benchmark regression model is specified as follows:

(2) $$\theta =\alpha_{i}+\sum \beta_{i}\cdot Scale_{i}+\sum \gamma_{i}\cdot Controls_{i}+\varepsilon_{i}$$

In Equation (2), θ represents the production efficiency of the farms. *Scale_i_* denotes the operating-scale group of *the i-th* pig farm and is introduced as a set of dummy variables. *β_i_* represents the corresponding regression coefficients of the operating-scale group dummy variables. *α_i_* is the intercept term, and *ε_i_* is the random error term, which is assumed to have a zero mean, constant variance, and no autocorrelation. *Controls_i_* denotes the control variables that may be associated with the production efficiency of pig farms. Considering that farm owner characteristics, farm characteristics, and external environmental conditions may be associated with the production efficiency of pig farms [47,48], this study includes a set of control variables. At the owner level, the model includes age, years of farming experience, education level, disease prevention training, government services, internet-based services, farming skills training, and market information services. At the farm level, farm labor, farm topography, farm site type, farming loans, and pig insurance are included. For external environmental conditions, surrounding disease risk is included as a control variable.

#### 2.3.2. Moderate Operating Scale from the Perspective of Disease Prevention and Control Performance

To further compare disease prevention and control performance across pig farms with different operating scales, this study modifies Equation (2) and specifies the disease prevention and control performance model as shown in Equation (3):

(3) $$W=\alpha_{i}+\sum \beta_{i}\cdot Scale_{i}+\sum \gamma_{i}\cdot Controls_{i}+\varepsilon_{i}$$

In Equation (3), the pig mortality rate (*W*) is used as the dependent variable. It is calculated as the ratio of the annual number of pig deaths to the sum of slaughtered pigs and pig deaths reported by the sampled pig farms. This indicator reflects the disease prevention and control outcome of pig farms under a given operating scale and management condition. To account for other factors that may be associated with disease prevention and control performance, the control variables are reselected based on Huang et al. and Chen et al. [49,50], as well as the actual survey conditions. At the owner level, the model includes age, years of farming experience, education level, disease prevention training, government services, and internet-based services. At the farm level, farm labor, farm topography, farm site type, farming loans, pig insurance, disease prevention investment, cleaning and disinfection frequency, and biosecurity level are included. For external environmental conditions, surrounding disease risk is included as a control variable. The meanings of the other variable symbols are the same as those in Equation (2).

#### 2.3.3. Moderate Operating Scale from the Perspective of Environmental Management Cost

To further compare the environmental management cost burden across pig farms with different operating scales, this study modifies Equation (2) and specifies the manure treatment cost model as shown in Equation (4):

(4) $$Y=\alpha_{i}+\sum \beta_{i}\cdot Scale_{i}+\sum \gamma_{i}\cdot Controls_{i}+\varepsilon_{i}$$

In Equation (4), the unit manure treatment cost (*Y*) of pig farms is used as the dependent variable. It refers specifically to the treatment cost per cubic meter of pig manure and urine, and reflects the actual environmental management cost borne by pig farms to meet environmental protection requirements under a given operating scale.

Considering that manure treatment methods, resource utilization conditions, and investment in treatment facilities may be associated with environmental management cost, this study reselects the control variables based on the existing literature [51,52,53,54], as well as the actual survey conditions. At the owner level, age, years of farming experience, education level, and internet-based services are included as control variables. At the farm level, farm labor, farm topography, farm site type, manure collection method, treatment facility investment, supporting cropland, self-use of biogas slurry, giving away biogas slurry, sale of biogas slurry, and manure treatment subsidy are included. The meanings of the other variable symbols are the same as those in Equation (2).

Given that the primary purpose of this study is to compare average differences in operational performance across operating-scale groups, rather than to construct a complete structural behavioral model, OLS is adopted as the baseline regression model. Under this specification, the estimated coefficients of the operating-scale group dummy variables can directly reflect the conditional mean differences between each operating-scale group and the reference group. This facilitates comparisons of operational performance across operating-scale groups. The 1–499 head group is used as the reference group, and this study focuses on the significance and magnitude of the coefficients of the operating-scale group dummy variables.

Furthermore, production efficiency and pig mortality rate are bounded between 0 and 1. It should be noted that the mortality rate reported in Supplementary Tables S1 and S2 and in the baseline regression results is expressed in percentage terms. This differs from a proportional representation only in scale and does not affect the substantive interpretation of the variable. In addition, unit manure treatment cost may exhibit a certain degree of skewness. Therefore, the production efficiency model and the disease prevention and control model are re-estimated using the Tobit model, while the environmental management cost model is re-estimated after taking the logarithm of manure treatment cost. These robustness checks are used to examine whether the main conclusions are sensitive to the estimation method and variable specification. In addition, considering that older farm owners may differ from other age groups in operational behavior, risk preference, and production organization, this study re-estimates the three models after excluding samples with farm owners aged over 60. Production efficiency was estimated using DEAP Version 2.1, while all descriptive statistics and regression analyses were performed using Stata Version 16.0.

## 3. Results

### 3.1. Benchmark Regression Results

Before parameter estimation, this study first performed VIF tests on all explanatory variables. The results show no multicollinearity in the production efficiency model, the disease prevention and control performance model, or the manure treatment cost model.

#### 3.1.1. Regression Results of the Production Efficiency Model

First, this study measured the production efficiency of pig farms using the DEA model. The results show that the average production efficiency of the full sample is 0.7449, with a standard deviation of 0.1262. The regression results in Table 1 report the signs, magnitudes, and statistical significance of the estimated coefficients. As shown in columns (1) and (2) of Table 1, regardless of whether control variables are included, the coefficients of all operating-scale group dummy variables are significant at the *p* < 0.01 (1%) level. Among them, the estimated coefficient of the 5000–10,000 head group is markedly higher than those of the other operating-scale groups. In addition, farming skills training and surrounding disease risk are significant at the *p* < 0.01 (1%) level, while market information services are significant at the *p* < 0.05 (5%) level.

#### 3.1.2. Regression Results of the Disease Prevention and Control Model

The regression results in Table 2 report the signs, magnitudes, and statistical significance of the estimated coefficients. As shown in columns (1) and (2) of Table 2, regardless of whether control variables are included, the coefficients of all operating-scale group dummy variables are significant at the *p* < 0.01 (1%) level. Among them, the estimated coefficient of the 3000–4999 head group is markedly lower than those of the other operating-scale groups. In addition, cleaning and disinfection frequency is significant at the *p* < 0.01 (1%) level. Disease prevention investment, farm site type, and surrounding disease risk are significant at the *p* < 0.05 (5%) level. Disease prevention training and farming loans are significant at the *p* < 0.10 (10%) level. Years of farming experience, education level, government services, internet-based services, farm labor, farm topography, pig insurance, and biosecurity level are not statistically significant.

#### 3.1.3. Regression Results of the Environmental Management Model

The regression results in Table 3 report the signs, magnitudes, and statistical significance of the estimated coefficients. As shown in columns (1) and (2) of Table 3, regardless of whether control variables are included, the coefficients of all operating-scale group dummy variables are significant at the *p* < 0.01 (1%) level. Among them, the estimated coefficient of the 3000–4999 head group is markedly lower than those of the other operating-scale groups. In addition, farm labor and sale of biogas slurry are significant at the *p* < 0.05 (5%) level. Age, years of farming experience, education level, internet-based services, farm topography, farm site type, manure collection method, treatment facility investment, supporting cropland, self-use of biogas slurry, giving away biogas slurry, and manure treatment subsidy are not statistically significant.

### 3.2. Robustness Test Results

This study uses the Tobit model to test the robustness of the regression results for the production efficiency model and the disease prevention and control model. The results are reported in columns (1) and (3) of Table 4. In addition, the environmental management model is re-estimated after taking the logarithm of manure treatment cost, and the results are reported in column (5) of Table 4. The results obtained after excluding samples with farm owners aged over 60 are reported in columns (2), (4), and (6) of Table 4. As shown in Table 4, after changing the estimation method, transforming the dependent variable, and excluding older farm owners from the sample, the signs and significance levels of the coefficients of the operating-scale group dummy variables remain broadly consistent with the baseline regression results. This confirms the robustness of the baseline results.

## 4. Discussion

This section interprets the empirical results within an integrated farm management and veterinary science framework. Production efficiency reflects the organizational and resource-use capacity of pig farms, which may provide the management basis for routine disease prevention and herd health practices. Pig mortality rate is interpreted as a farm-level animal health and epidemiological indicator, while manure treatment cost is discussed as an environmental management indicator with One Health implications. Therefore, the moderate operating scale of pig farming is assessed not only as an economic or managerial issue, but also as a question related to herd health management, disease-risk control, and environmental management.

As shown by the regression results of the production efficiency model in Table 1, with the 1–499 head group as the reference group, the coefficients of the other four operating-scale groups are all positive. This indicates that, after controlling for other variables, these groups show higher production efficiency than the reference group to varying degrees. The estimated coefficients of these groups are all positive and are ranked as follows: 500–1499 head < 1500–2999 head < 3000–4999 head < 5000–10,000 head. Among them, the coefficient of the 5000–10,000 head group is markedly higher than those of the other operating-scale groups. This suggests that this group shows the most pronounced production efficiency advantage relative to the 1–499 head group. Therefore, based on the current sample survey, the 5000–10,000 head group can be regarded as the relatively better-performing group from the perspective of production efficiency, while the 3000–4999 head group also shows relatively strong production efficiency performance. This result indicates that larger operating scales may be associated with stronger resource allocation and production organization capacity. In pig farming, better coordination of feed use, labor allocation, production scheduling, and routine management can help maintain production stability and provide a management basis for routine disease prevention and herd health practices.

The regression results in Table 1 further show that the coefficients of farming skills training and market information services are significantly positive. This indicates that these variables are associated with higher production efficiency among pig farms. Participation in farming skills training and access to market information services may help farm owners improve production technology, obtain market information in a timely manner, and optimize production organization and business decision-making. At the same time, farm owners with access to these services may also have stronger willingness and ability to learn, which is related to higher production efficiency. From a broader management perspective, these factors may also improve the capacity of farm owners to organize daily production and respond to operational risks. This finding is consistent with Manevska-Tasevska et al. [55], who reported that greater accumulation of human capital and management skills among livestock farm owners is conducive to sustained efficiency improvement. The coefficient of surrounding disease risk is also significant. This suggests that pig farms facing lower surrounding disease risk tend to have higher production efficiency. From a veterinary epidemiological perspective, surrounding disease risk represents an external health-risk environment faced by pig farms. Lower external disease risk may help reduce production disruption, mortality losses, and additional prevention costs, thereby stabilizing the production process and supporting higher production efficiency. This finding is consistent with Piao et al. [56], who reported that African swine fever significantly reduced the overall production efficiency of China’s pig farming industry. Their results suggest that lower external epidemic risk is important for reducing production losses and maintaining stable production.

As shown by the regression results of the disease prevention and control model in Table 2, with the 1–499 head group as the reference group, the coefficients of the other four operating-scale groups are all negative. This indicates that, after controlling for other variables, these groups show lower pig mortality rates than the reference group to varying degrees. Pig mortality rate is not only a production-loss indicator, but also an important farm-level indicator of animal health status and epidemiological risk. Although it does not directly measure disease incidence, pathogen infection, or specific clinical conditions, it captures the final health losses associated with disease occurrence, management failure, environmental stress, and delayed or ineffective intervention. Therefore, differences in pig mortality across operating-scale groups provide useful evidence for evaluating herd health outcomes and disease-risk management capacity under different farm scales. The absolute values of the estimated coefficients are ranked as follows: 5000–10,000 head < 500–1499 head < 1500–2999 head < 3000–4999 head. Among them, the 3000–4999 head group shows the largest reduction in pig mortality relative to the 1–499 head group, suggesting relatively better disease prevention and control performance and stronger herd health management capacity. From an animal health perspective, this finding suggests that the 3000–4999 head group may be more capable of reducing avoidable health losses and maintaining a relatively stable herd health status. When the annual slaughter volume exceeds 5000 head, the reduction in pig mortality relative to the reference group becomes smaller. This suggests that, as operating scale continues to expand, the increasing complexity of disease prevention and control may offset part of the improvement in prevention and control capacity associated with scale expansion. Therefore, based on the current sample, the 3000–4999 head group can be regarded as the relatively better-performing group from the perspective of disease prevention and control.

This result has important implications for understanding the relationship between farm scale, herd health management, and veterinary intervention. The lower pig mortality rate observed in the 3000–4999 head group may reflect a more favorable balance between disease-control resources and management complexity. Farms in this range may have sufficient capacity to invest in disease prevention facilities, implement regular cleaning and disinfection, maintain relatively standardized biosecurity routines, and obtain veterinary or epidemic-prevention services, while still keeping herd organization and daily health monitoring within a manageable range. In this sense, the relative advantage of this scale range may lie not only in greater access to disease-control resources, but also in the ability to translate these resources into effective and timely herd health management [57]. However, when the operating scale becomes very large, disease prevention and control may face a different set of epidemiological challenges. Scale expansion usually means more pigs, more production units or pens, more frequent movement of pigs, feed, vehicles, personnel, and manure, and more contact points between the farm and the external environment. Each of these contacts may become a potential pathway for pathogen introduction or within-farm transmission if biosecurity protocols are not implemented consistently. Lambert et al. found that pathogen introduction into swine herds may occur through contacts involving people, animals, vehicles, and supplies, and that larger herd size was associated with more frequent staff, visitor, and live-pig movements [58]. Galvis and Machado further showed that vehicles transporting feed, pigs, and people can create extensive contact networks among swine farms, highlighting the importance of vehicle movement and cleaning effectiveness in disease dissemination [59]. Martinez et al. also emphasized that continuous pig movement in multi-site pig production systems exposes farms to animals with different health statuses, as well as to personnel, supplies, and equipment from different locations [60].

Therefore, the weaker mortality-reduction advantage observed in the 5000–10,000 head group should not be interpreted as a simple failure of large-scale production. Rather, it may indicate that, beyond a certain scale, the health benefits of stronger disease-control resources can be partly offset by higher exposure frequency, more complex contact networks, and greater difficulty in maintaining consistent biosecurity implementation. The effectiveness of biosecurity measures depends on consistent implementation across the entire prevention chain. As scale increases, any weak point in this chain—including disinfection, isolation, entry control, pig-flow management, and the movement of personnel, vehicles, materials, and animals—may reduce overall biosecurity effectiveness and increase the possibility of pathogen entry or circulation within the herd. From a veterinary management perspective, larger farms require more demanding herd health strategies, including stricter entry control, vehicle cleaning and disinfection, pig-flow management, early disease detection, isolation of abnormal animals, and timely veterinary intervention. Evidence from China also suggests that medium and large pig farms may face higher PRRSV infection pressure than small farms, possibly because larger farms involve more frequent pig-to-pig contact and more complex herd structures [61]. In the cold climatic context of Heilongjiang Province, winter environmental control may further intensify the management complexity of very large pig farms. During winter, pig houses need to maintain adequate ventilation to reduce humidity, harmful gases, airborne particles, and pathogen accumulation, while also preserving indoor temperature and avoiding cold stress. This creates a practical trade-off among ventilation, heating, and disinfection. If ventilation is reduced to maintain warmth, air quality may deteriorate and pathogen pressure may increase; if ventilation is increased, greater heating demand and temperature fluctuations may occur, which may weaken herd health stability [62,63]. In addition, low-temperature conditions may increase the difficulty of routine cleaning and disinfection, particularly when surfaces, vehicles, or manure-related areas are difficult to dry sufficiently. These challenges may be more pronounced in very large farms because more barns, animals, personnel, vehicles, and manure-handling points need to be managed simultaneously. Therefore, under the cold winter conditions of Heilongjiang, the coordination of ventilation, heating, disinfection, and biosecurity implementation may be an additional contextual mechanism through which very large operating scales face higher herd health management difficulty. These mechanisms help explain why the 3000–4999 head group may achieve a better balance between biosecurity capacity and epidemiological risk exposure in the current sample.

Consistent with this mechanism, the regression results in Table 2 further show that disease prevention training, disease prevention investment, cleaning and disinfection frequency, and surrounding disease risk are significantly associated with pig mortality. Together, these variables reflect different components of herd health management, including disease-risk awareness, preventive resource allocation, routine biosecurity implementation, and external epidemiological pressure. These findings are broadly consistent with previous evidence on the role of training and biosecurity management in pig production. Dione et al. [64] showed that a deeper understanding of disease-related knowledge can help farmers improve biosecurity practices and disease prevention outcomes. Laanen et al. [65] found that higher levels of on-farm biosecurity and hygiene management were associated with lower disease occurrence and health losses. In this study, farm owners with more professional knowledge of disease prevention may be more likely to identify disease risks at an earlier stage, invest in disease prevention facilities and supplies, strengthen routine cleaning and disinfection, and maintain a more stable production environment. These factors may help reduce mortality losses and improve herd health outcomes. In addition, pig farms with farming loans show higher pig mortality rates than those without loans. This finding is consistent with Li et al. [66], but differs from Ebata et al. [67]. A possible explanation is that farming loans can help meet capital needs in pig production, but they may also impose repayment obligations and cash-flow constraints. Under heavier financial pressure, pig farms may reduce investment in disease prevention and control or lower their management requirements, which may weaken routine health management and thus be associated with higher pig mortality.

As shown by the regression results of the environmental management model in Table 3, with the 1–499 head group as the reference group, the coefficients of the other four operating-scale groups are all negative. This indicates that, after controlling for other variables, these groups show lower unit manure treatment costs than the reference group to varying degrees. The absolute values of the estimated coefficients are ranked as follows: 5000–10,000 head < 1500–2999 head < 500–1499 head < 3000–4999 head. Among them, the 3000–4999 head group shows the largest reduction in unit manure treatment cost relative to the 1–499 head group, suggesting the strongest cost-control advantage. However, as operating scale continues to expand, expenditures on farm maintenance, facility operation, manure transport, and subsequent utilization may also increase, thereby narrowing the cost advantage. Therefore, based on the current sample, the 3000–4999 head group can be regarded as the relatively better-performing group from the perspective of environmental management cost.

The regression results in Table 3 further show that farm labor and sale of biogas slurry are significantly associated with lower manure treatment costs. A possible explanation is that, when labor is relatively sufficient, manure cleaning, collection, transport, and routine treatment can be carried out more promptly. This may improve treatment efficiency and lower unit manure treatment cost. In addition, the sale of biogas slurry, as a market-oriented utilization approach, may help alleviate expenditure pressure associated with manure treatment. These findings are consistent with the broader economic mechanisms identified in previous manure-management studies. Petersen et al. [68] reported that internal farm management and resource allocation are important for controlling manure treatment costs. Vingerhoets et al. [69] found that value-added treatment of manure-derived products can reduce manure disposal costs.

Manure management is a key point at which pig production, farm environmental hygiene, animal health, and public health intersect. In pig production systems, manure and wastewater are generated continuously. If manure collection, storage, treatment, and utilization are not conducted in a timely and standardized manner, the farm environment may become a reservoir for pathogenic microorganisms, including bacteria and viruses, thereby increasing the risk of environmental re-exposure and within-herd transmission [70]. Poorly managed manure may also allow pathogens, antimicrobial-resistant bacteria, and other health-related contaminants to enter soil and water systems, creating potential public health concerns at the animal–environment–human interface [71]. Therefore, manure handling should be viewed not only as an environmental compliance task, but also as an important measure for reducing opportunities for pathogen circulation at the animal–environment–human interface and as a component of internal biosecurity and preventive veterinary management [32,37,72].

In this study, unit manure treatment cost does not directly measure pathogen loads, antimicrobial residues, or public health outcomes. Rather, it reflects the cost burden and organizational efficiency of manure collection, treatment, and resource utilization under different operating scales. The finding that the 3000–4999 head group shows the strongest cost-control advantage suggests that this scale range may have a relatively stronger capacity to organize manure treatment and resource utilization efficiently. From a One Health-oriented interpretation, such environmental management capacity is relevant because better-organized manure handling can help maintain farm environmental hygiene, support biosecurity implementation, reduce potential environmental pathogen pressure, and lower health-related risks at the animal–environment–human interface. Therefore, the environmental management dimension of this study provides an important supplementary perspective for understanding the moderate operating scale of pig farming in relation to animal health, disease prevention, and One Health-oriented risk management.

Based on the above analysis, after controlling for other variables, the operating-scale groups show different relative performance across the three dimensions. From the perspective of production efficiency, the estimated coefficients, all of which are positive, are ranked as follows: 1–499 head < 500–1499 head < 1500–2999 head < 3000–4999 head < 5000–10,000 head. From the perspective of disease prevention and control performance, the absolute values of the estimated coefficients, all of which are negative, are ranked as follows: 1–499 head < 5000–10,000 head < 500–1499 head < 1500–2999 head < 3000–4999 head. From the perspective of environmental management cost, the absolute values of the estimated coefficients, all of which are negative, are ranked as follows: 1–499 head < 5000–10,000 head < 1500–2999 head < 500–1499 head < 3000–4999 head. These results indicate that the assessment of moderate operating scale should not rely solely on production efficiency. From a veterinary science perspective, pig mortality provides evidence on herd health outcomes and epidemiological risk management, while manure treatment cost reflects the capacity of pig farms to organize manure management, which is relevant to farm environmental hygiene, animal health risks, and potential health concerns at the animal–environment–human interface. If only production efficiency is considered, the relatively better-performing operating-scale group tends to be larger. However, after animal health outcomes, disease prevention capacity, and environmental management are further considered, the relatively suitable operating-scale range becomes smaller. Overall, under the current conditions of pig farming in Heilongjiang Province, the 3000–4999 head group shows stronger coordination across production efficiency, pig mortality reduction, and manure treatment cost control. It may therefore represent a relatively suitable operating-scale range for balancing production performance, herd health management, and environmental management.

Several limitations should be considered when interpreting these findings. First, the study was conducted among specialized fattening pig farms in Heilongjiang Province, where climatic conditions, production organization, disease-risk environments, and environmental-management conditions may differ from those in other regions or production categories. Second, the analysis relied on cross-sectional and partly self-reported farm data, which may be subject to recall and reporting bias. The cross-sectional design also cannot capture long-term changes in farm performance and limits causal interpretation. Third, although restricting the sample to specialized fattening pig farms improved comparability across production units, the analytical unit was the pig farm rather than the individual animal or production batch. Consequently, some variation in animal and batch characteristics, such as piglet source, entry weight, and batch composition, could not be fully captured by the farm-level survey. Finally, pig mortality was used as a general indicator of disease prevention and control performance rather than as a disease-specific epidemiological measure. Future studies incorporating longitudinal data, animal- and batch-level characteristics, disease-specific indicators, and market accessibility variables would provide a more comprehensive understanding of the relationship between operating scale and farm performance.

## 5. Conclusions

This study examines the moderate operating scale of pig farming in Heilongjiang Province from three dimensions: production efficiency, disease prevention and control, and environmental management. The results show that the relatively better-performing operating-scale group differs across evaluation dimensions. The specific findings are as follows:

- From the perspective of production efficiency, the 5000–10,000 head group shows the strongest relative performance among the operating-scale groups.
- From the perspective of animal health and disease prevention, the 3000–4999 head group shows the strongest relative performance.
- From the perspective of environmental management cost, the 3000–4999 head group shows the strongest cost-control advantage.

Based on these findings, there are trade-offs among improving production efficiency, reducing pig mortality, and controlling environmental management costs in Heilongjiang’s pig farming sector. Therefore, the moderate operating scale of pig farming should not be assessed solely from the perspective of economic efficiency. Animal health outcomes, herd-level disease prevention capacity, biosecurity implementation, and environmental risks should also be considered. Overall, under the current conditions of pig farming in Heilongjiang Province, an annual slaughter volume of 3000–4999 head may represent a relatively suitable operating-scale range for balancing production performance, herd health management, and environmental management. These findings may provide useful evidence for scale guidance, disease-risk management, and sustainable pig production in regions with characteristics similar to those of Heilongjiang Province.

## Figures and Tables

**Table 1:** Regression results of the production efficiency model for pig farms.

Variable	(1)	(2)	Variable	(1)	(2)
	Production Efficiency	Production Efficiency		Production Efficiency	Production Efficiency
S2	0.0352 ***	0.0389 ***	FST		0.0763 ***
	(0.0116)	(0.0121)			(0.0140)
S3	0.0689 ***	0.0785 ***	MIS		0.0199 **
	(0.0150)	(0.0165)			(0.0089)
S4	0.0832 ***	0.0891 ***	FL		−0.0024
	(0.0155)	(0.0196)			(0.0032)
S5	0.0880 ***	0.1140 ***	FT		−0.0025
	(0.0170)	(0.0247)			(0.0057)
AGE		0.0006	SITE		−0.0146
		(0.0006)			(0.0233)
EXP		0.0007	LOAN		−0.0001
		(0.0007)			(0.0088)
EDU		−0.0009	INS		−0.0058
		(0.0018)			(0.0106)
DPT		−0.0118	SDR		0.0206 ***
		(0.0101)			(0.0065)
GS		−0.0117	Const.	0.7180 ***	0.5949 ***
		(0.0119)		(0.0056)	(0.0509)
IBS		−0.0063	N	809	809
		(0.0118)	R^2^	0.067	0.120

Note: Standard errors are shown in parentheses. *** and ** indicate statistical significance at the 1% and 5% levels, respectively. Abbreviations: S2, annual slaughter volume of 500–1499 head; S3, annual slaughter volume of 1500–2999 head; S4, annual slaughter volume of 3000–4999 head; S5, annual slaughter volume of 5000–10,000 head; AGE, age; EXP, years of farming experience; EDU, education level; DPT, disease prevention training; GS, government services; IBS, internet-based services; FST, farming skills training; MIS, market information services; FL, farm labor; FT, farm topography; SITE, farm site type; LOAN, farming loans; INS, pig insurance; SDR, surrounding disease risk.

**Table 2:** Regression results of the disease prevention and control model for pig farms.

Variable	(1)	(2)	Variable	(1)	(2)
	Pig Mortality Rate	Pig Mortality Rate		Pig Mortality Rate	Pig Mortality Rate
S2	−2.1087 ***	−1.9456 ***	FT		−0.1722
	(0.2050)	(0.2085)			(0.1561)
S3	−2.3976 ***	−2.1842 ***	SITE		−0.6983 **
	(0.1926)	(0.2301)			(0.2895)
S4	−2.4340 ***	−2.2624 ***	DPI		−0.0218 **
	(0.1889)	(0.2841)			(0.0101)
S5	−2.4239 ***	−1.5520 ***	CDF		−0.3759 ***
	(0.1863)	(0.4156)			(0.1284)
AGE		−0.0064	LOAN		0.3727 *
		(0.0140)			(0.2085)
EXP		−0.0122	INS		0.0918
		(0.0190)			(0.2584)
EDU		−0.0496	BSL		−0.2083
		(0.0414)			(0.1270)
DPT		−0.3921 *	SDR		−0.2074 **
		(0.2368)			(0.1019)
GS		0.0457	Const.	2.5557 ***	6.3139 ***
		(0.3156)		(0.1853)	(1.3811)
IBS		0.5084	N	809	809
		(0.3540)	R^2^	0.135	0.177
FL		−0.0224			
		(0.0692)			

Note: Standard errors are shown in parentheses. ***, **, and * indicate statistical significance at the 1%, 5%, and 10% levels, respectively. Abbreviations: S2, annual slaughter volume of 500–1499 head; S3, annual slaughter volume of 1500–2999 head; S4, annual slaughter volume of 3000–4999 head; S5, annual slaughter volume of 5000–10,000 head; AGE, age; EXP, years of farming experience; EDU, education level; DPT, disease prevention training; GS, government services; IBS, internet-based services; FL, farm labor; FT, farm topography; SITE, farm site type; DPI, disease prevention investment; CDF, cleaning and disinfection frequency; LOAN, farming loans; INS, pig insurance; BSL, biosecurity level; SDR, surrounding disease risk.

**Table 3:** Regression results of the environmental management model for pig farms.

Variable	(1)	(2)	Variable	(1)	(2)
	Manure Treatment Cost	Manure Treatment Cost		Manure Treatment Cost	Manure Treatment Cost
S2	−3.7512 ***	−3.4984 ***	SITE		0.2226
	(0.2714)	(0.2948)			(0.4878)
S3	−3.6704 ***	−3.4132 ***	MCM		0.0218
	(0.1327)	(0.1963)			(0.1104)
S4	−4.6300 ***	−4.2322 ***	TFI		0.0012
	(0.1727)	(0.2775)			(0.0017)
S5	−2.7412 ***	−1.9403 ***	SC		0.0032
	(0.4261)	(0.5530)			(0.2046)
AGE		−0.0120	SBS		0.2781
		(0.0114)			(0.2107)
EXP		−0.0164	GBS		0.2709
		(0.0139)			(0.1926)
EDU		0.0398	SALBS		−1.0288 **
		(0.0365)			(0.4516)
IBS		−0.0833	MTS		−0.1978
		(0.2386)			(0.1823)
FL		−0.1443 **	Const.	15.9473 ***	15.8872 ***
		(0.0649)		(0.1154)	(0.8343)
FT		0.0902	N	809	809
		(0.1245)	R^2^	0.383	0.398

Note: Standard errors are shown in parentheses. *** and ** indicate statistical significance at the 1% and 5% levels, respectively. Abbreviations: S2, annual slaughter volume of 500–1499 head; S3, annual slaughter volume of 1500–2999 head; S4, annual slaughter volume of 3000–4999 head; S5, annual slaughter volume of 5000–10,000 head; AGE, age; EXP, years of farming experience; EDU, education level; IBS, internet-based services; FL, farm labor; FT, farm topography; SITE, farm site type; MCM, manure collection method; TFI, treatment facility investment; SC, supporting cropland; SBS, self-use of biogas slurry; GBS, giving away biogas slurry; SALBS, sale of biogas slurry; MTS, manure treatment subsidy.

**Table 4:** Robustness test.

Variable	Production Efficiency		Pig Mortality Rate		Manure Treatment Cost	
	(1)	(2)	(3)	(4)	(5)	(6)
S2	0.0416 ***	0.0376 ***	−0.0195 ***	−1.8981 ***	−0.2718 ***	−3.6428 ***
	(0.0127)	(0.0127)	(0.0021)	(0.2188)	(0.0229)	(0.3084)
S3	0.0819 ***	0.0873 ***	−0.0218 ***	−2.1126 ***	−0.2297 ***	−3.4627 ***
	(0.0174)	(0.0168)	(0.0023)	(0.2444)	(0.0143)	(0.2055)
S4	0.0957 ***	0.0975 ***	−0.0226 ***	−2.1987 ***	−0.3015 ***	−4.3748 ***
	(0.0212)	(0.0207)	(0.0028)	(0.3035)	(0.0212)	(0.2965)
S5	0.1220 ***	0.1205 ***	−0.0155 ***	−1.5692 ***	−0.1317 ***	−2.1473 ***
	(0.0263)	(0.0254)	(0.0041)	(0.4329)	(0.0420)	(0.5671)
Const.	0.5866 ***	0.5715 ***	0.0631 ***	7.1810 ***	2.7541 ***	16.1061 ***
	(0.0541)	(0.0551)	(0.0136)	(1.4320)	(0.0606)	(0.9032)
Control Variables	Controlled	Controlled	Controlled	Controlled	Controlled	Controlled
N	809	735	809	735	809	735
Pseudo R^2^/R^2^	−0.1290	0.131	−0.0473	0.172	0.401	0.414

Note: Standard errors are shown in parentheses. *** indicate statistical significance at the 1% levels.

## Data Availability

The original contributions presented in this study are included in the article/Supplementary Material. Further inquiries can be directed to the corresponding author.

## Supplementary Materials

The following supporting information can be downloaded at https://www.mdpi.com/article/10.3390/vetsci13080728/s1: Supplementary Material File S1, English questionnaire used in the field survey; Supplementary Material File S2, Definitions and descriptive statistics of variables used in the empirical analysis, Table S1, Categorical variables; Table S2, Continuous variables.
