# Supplementary material for: Moderate Scale of Pig Farming: Implications for Herd Management and Disease Control—Evidence from Heilongjiang Province, China
Source: Vet Sci. 2026 Jul 24;13(8):728. doi: 10.3390/vetsci13080728 (PMC13517646; doi:10.3390/vetsci13080728)
Supplement: Supplementary file 1 [file vetsci-13-00728-s001.zip › Supplementary Material File S1.pdf]

Questionnaire for Specialized Fattening Pig Farms

Promoting the development of moderately scaled pig farming is of great importance for the high-quality and sustainable development of China's pig industry. To support this objective, the Livestock Economics Innovation Team of the Institute of Animal Sciences, Chinese Academy of Agricultural Sciences, conducted this survey.[1]

This questionnaire aims to gain a comprehensive understanding of the current production and management practices, environmental management investments, and disease prevention and control strategies of specialized fattening pig farms. Your responses are extremely valuable to this study and will provide important practical evidence for exploring sustainable pathways for the future development of the pig industry.

All information collected through this survey will be kept strictly confidential and used solely for academic research purposes.

Thank you very much for your time and support!

Part I. Farm and Farmer Characteristics

Q1. Farm location

Province: ____________ City: ____________

Q2. What is the predominant topographical feature of the area where your farm is located?

○ Plain/Basin (flat and open terrain)

○ Hilly area (gently undulating terrain with moderate slopes)

○ Mountainous area (rugged terrain with steep slopes and deep valleys)

Q3. What is your year of birth? (e.g., 1970)

____

Q4. What is your highest level of education?

○ Primary school

○ Junior high school

○ Senior high school / technical secondary school

○ Junior college

○ Bachelor's degree

○ Graduate degree or above

Q5. How many years have you been engaged in pig farming?

____________________________ years

Q6. Labor information

Total number of workers on the farm: ________ persons; number of hired workers: ________ persons; total annual labor cost: ________ (10,000 RMB/year).

Q7. What is the current cash reserve status of your farm?

○ Extremely tight; difficult to maintain normal operations

○ Tight; sufficient only for the next 1-3 months of operation

○ Adequate; sufficient for the next 3-6 months of operation

○ Sufficient; sufficient for more than 6 months of operation

[1] Note: This document is an English translation of the questionnaire used during the field survey.

Q8. Did your farm have any loans in 2024?

○ Yes

○ No

Q9. If your farm had loans in 2024, approximately how much was the loan amount?

○ Less than 100,000 RMB

○ 100,000-500,000 RMB

○ 500,000-1,000,000 RMB

○ More than 1,000,000 RMB

Q10. Did your farm participate in pig insurance programs in 2024?

○ Yes

○ No

Q11. Did you receive services from internet-based service platforms in 2024?

○ Yes

○ No

Q12. Did you receive services from universities or research institutions in 2024?

○ Yes

○ No

Q13. Did you receive services from government veterinary stations or disease prevention stations in 2024?

○ Yes

○ No

Q14. Did you receive services from private veterinary clinics in 2024?

○ Yes

○ No

Q15. Did you receive training in pig production technology, feed nutrition, or feed formulation consulting services in 2024?

○ Yes

○ No

Q16. Did you receive market information services in 2024?

○ Yes

○ No

Q17. Did you receive industry policy interpretation services in 2024?

○ Yes

○ No

Q18. Did you receive professional training in disease prevention and control in 2024?

○ Yes

○ No

Q19. Pig sales in 2024

Number of pigs sold during 2024: ________ head; total sales revenue: ________ (10,000 RMB).

Q20. What is the ownership status of your farm?

○ Self-owned farm

○ Leased farm

For self-owned farms:

Annual land rent: ________ (10,000 RMB/year; enter 0 if none); investment in farm buildings and infrastructure (e.g., fattening barns): ________ (10,000 RMB); investment in equipment (e.g., ventilation systems and air-conditioning systems): ________ (10,000 RMB).

For leased farms:

Annual rent: ________ (10,000 RMB/year).

Have you made additional investments in facilities or equipment (e.g., facility renovation or equipment purchase)?

○ Yes (please specify the total investment): ________ (10,000 RMB)

○ No

Q21. Herd size information

Designed herd capacity of the farm: ________ head.

Actual inventory at the end of December 2023 (including piglets and fattening pigs): ________ head.

Actual inventory at the end of December 2024 (including piglets and fattening pigs): ________ head (enter 0 if none).

Current inventory at the time of the survey (including piglets and fattening pigs): ________ head (enter 0 if none).

Part II. Production Inputs and Performance

Q22. Feed consumption and feed cost

Item	2024
Feed consumption per fattening pig (kg/head)	
Feed cost per fattening pig (RMB/head)	

Q23. Other production costs of the farm (enter 0 if none)

Item	2024
Equipment maintenance cost (10,000 RMB/year; expenses for hiring technicians to repair equipment)	
Fuel and power cost (10,000 RMB/year)	
Veterinary and disease prevention cost (including veterinary services, medicines, and vaccines) (10,000 RMB/year)	
Technical consulting service cost (10,000 RMB/year)	
Water cost (10,000 RMB/year)	
Electricity cost (10,000 RMB/year)	
Other costs (RMB/year)	

Q24. Production Input and Output Indicators

Item	2024
Average weight of purchased piglets (kg/head)	
Total number of purchased piglets (head/year)	
Average purchase price of piglets (RMB/head)	
Average market weight of fattening pigs sold (kg/head)	
Average selling price of fattening pigs (RMB/kg)	
Average production cost per pig marketed (RMB/head)	
Average profit per pig marketed (RMB/head)	

Part III. Disease Prevention and Control

Q25. Pig mortality in 2024

Number of pigs that died in 2024: ________ head; number of piglet deaths in 2024: ________ head; number of fattening pig deaths in 2024: ________ head.

Q26. Please estimate the total investment in disease prevention and biosecurity facilities. ________ (10,000 RMB)

Q27. Which facilities are currently used on your farm to prevent and control African Swine Fever (ASF)?

□ Farm layout biosecurity measures (e.g., separation of clean and dirty pathways, isolation units)

□ Well-equipped intelligent farming facilities (e.g., health-monitoring devices, automated feeding systems, and other labor-saving automation equipment)

□ Complete disinfection facilities (e.g., disinfection passages, disinfectant pools, sprayers, vehicle cleaning and disinfection facilities)

□ Hardened internal roads

□ Reinforcement of farm infrastructure to prevent birds and rodents

□ Other (please specify): ____________________

Q28. What measures has your farm adopted to prevent and control major swine diseases (e.g., African Swine Fever)?

□ Strict control and isolation of personnel entering the farm

□ Increased frequency of cleaning and disinfection of vehicles, personnel, and materials entering the farm

□ Increased frequency of vacancy-period disinfection and routine disinfection

□ Quarantine and isolation of introduced pigs

□ Enhanced inspection and disinfection of feed

□ Safe disposal of dead pigs

□ Professional training in disease prevention and control

□ Other (please specify): ____________________

Q29. Please estimate the annual expenditure associated with the disease prevention and biosecurity measures adopted on your farm.________ (10,000 RMB/year)

Q30. How often do you conduct comprehensive cleaning and disinfection of the farm environment (e.g., floors and walls)?

○ Irregularly or rarely

○ Once per month

○ Once per week

○ Every 2-3 days

○ Every day

Q31. What was your annual expenditure on disinfectants?

________ (10,000 RMB/year)

Q32. Vaccination practices

Vaccination status in 2024: number of vaccine types administered per pig: ________; average vaccination cost per pig: ________ (RMB/head).

Please recall the vaccination status before the ASF outbreak (2017): number of vaccine types administered per pig: ________; average vaccination cost per pig: ________ (RMB/head).

Examples of vaccines include: Classical Swine Fever (CSF), Porcine Reproductive and Respiratory Syndrome (PRRS), Foot-and-Mouth Disease (FMD), Porcine Circovirus (PCV), Pseudorabies, Transmissible Gastroenteritis (TGE) and Porcine Epidemic Diarrhea (PED) bivalent (or trivalent) vaccines, Mycoplasma hyopneumoniae, Japanese Encephalitis, and others. Please enter only the number of vaccine types administered.

Q33. Please assess the level of investment made by your farm in disease surveillance and diagnostic testing (e.g., sample submission and antibody testing).

○ No disease testing is conducted

○ Testing is conducted only occasionally when disease occurrence is suspected

○ Routine disease testing is conducted once or twice per year

○ Routine disease testing is conducted once every quarter

○ Key indicators are monitored monthly or weekly with detailed records maintained

Q34. Please assess the availability of professional personnel for disease prevention and control on your farm.

○ No professional guidance or support has been obtained

○ Occasional professional guidance is obtained, but farm management mainly relies on self-management

○ Professional assistance is actively sought when disease outbreaks occur

○ Regular communication with professionals is maintained for routine guidance and recommendations

○ Long-term cooperation with professional personnel who are deeply involved in farm disease prevention and control activities

Q35. Approximately how far is your farm from the nearest pig farm of a similar type?

○ Less than 5 km

○ 5-10 km

○ 10-50 km

○ More than 50 km

Q36. How frequently did disease outbreaks occur among neighboring pig farms in 2024?

Examples include Porcine Epidemic Diarrhea (PED), Porcine Reproductive and Respiratory Syndrome (PRRS), African Swine Fever (ASF), and other major swine diseases.

○ Frequently

○ Occasionally

○ Rarely

○ Almost never

○ Never

Q37. Please rate the overall biosecurity management level of your farm.

Very low ○ 1 ○ 2 ○ 3 ○ 4 ○ 5 Very high

Part IV. Manure Management

Q38. What manure collection method is used on your farm? *Please complete the corresponding section according to the manure collection method used on your farm.*

○ Water-flushing system

○ Slurry pit system

○ Dry manure removal system

Section A. Farms Using Dry Manure Removal Systems

Q39. Please estimate the total investment in manure treatment facilities (e.g., urine storage tanks and composting sites).

________ (10,000 RMB)

Q40. Please estimate the manure treatment cost per cubic meter of pig manure and wastewater, including collection, treatment, transportation, land application, labor, equipment maintenance, water, and electricity.

________ RMB/m^3

Q41. Does your farm have supporting cropland available for manure utilization?

○ Yes (please specify the area: ________ mu)

○ No

Q42. How are biogas slurry and biogas residue utilized? *(Multiple-choice）*

If more than one option is selected, please indicate the percentage allocated to each option. The percentages should sum to 100%.

□ Self-use (percentage: ________%)

□ Provided free of charge to others (percentage: ________%)

□ Sold (percentage: ________%)

Q43. If biogas slurry or residue is provided free of charge, how is it handled?

○ Direct cooperation with crop farmers

○ Processed through a third-party organization

Q44. If biogas slurry or residue is sold, how is it handled?

○ Direct cooperation with crop farmers

○ Processed through a third-party organization

Q45. Are there any costs associated with providing biogas slurry or residue free of charge (e.g., transportation, labor, and fuel)?

○ Yes (please specify annual expenditure: ________ 10,000 RMB)

○ No

Q46. Are there any costs or revenues associated with selling biogas slurry or residue? *(Multiple-choice)*

□ Yes, costs incurred (please specify annual expenditure: ________ 10,000 RMB)

□ Yes, revenue generated (please specify annual sales revenue: ________ 10,000 RMB)

□ No costs and no revenues

Q47. Did your farm receive any government subsidies related to manure management? *(Multiple-choice)*

□ Subsidies for manure treatment facilities (e.g., urine storage tanks, composting sites, and other treatment or storage facilities) (amount: ________ 10,000 RMB)

□ Subsidies for manure treatment equipment (e.g., compost turners, solid-liquid separators, manure scrapers, and other manure treatment equipment) (amount: ________ 10,000 RMB)

□ Subsidies for manure treatment operations (amount: ________ 10,000 RMB)

□ No

Section B. Farms Using Slurry Pit Systems

Q48. Please estimate the total investment in manure treatment facilities (e.g., urine storage tanks and composting sites).

________ (10,000 RMB)

Q49. Please estimate the manure treatment cost per cubic meter of pig manure and wastewater, including collection, treatment, transportation, land application, labor, equipment maintenance, water, and electricity. *[Open-ended]*

________ RMB/m^3

Q50. Does your farm have supporting cropland available for manure utilization?

○ Yes (please specify the area: ________ mu)

○ No

Q51. How are biogas slurry and biogas residue utilized? *(Multiple-choice)*

If more than one option is selected, please indicate the percentage allocated to each option. The percentages should sum to 100%.

□ Self-use (percentage: ________%)

□ Provided free of charge to others (percentage: ________%)

□ Sold (percentage: ________%)

Q52. If biogas slurry or residue is provided free of charge, how is it handled?

○ Direct cooperation with crop farmers

○ Processed through a third-party organization

Q53. If biogas slurry or residue is sold, how is it handled?

○ Direct cooperation with crop farmers

○ Processed through a third-party organization

Q54. Are there any costs associated with providing biogas slurry or residue free of charge (e.g., transportation, labor, and fuel)? *[Single-choice]*

○ Yes (please specify annual expenditure: ________ RMB)

○ No

Q55. Are there any costs or revenues associated with selling biogas slurry or residue?*(Multiple-choice)*

□ Yes, costs incurred (please specify annual expenditure: ________ RMB)

□ Yes, revenue generated (please specify annual sales revenue: ________ RMB)

□ No costs and no revenues

Q56. Did your farm receive any government subsidies related to manure management? *(Multiple-choice)*

□ Subsidies for manure treatment facilities (e.g., urine storage tanks, composting sites, and other treatment or storage facilities) (amount: ________ 10,000 RMB)

□ Subsidies for manure treatment equipment (e.g., compost turners, solid-liquid separators, manure scrapers, and other manure treatment equipment) (amount: ________ 10,000 RMB)

□ Subsidies for manure treatment operations (amount: ________ 10,000 RMB)

□ No

Section C. Farms Using Water-Flushing Systems

Q57. Please estimate the total investment in manure treatment facilities (e.g., urine storage tanks and composting sites).

________ (10,000 RMB)

Q58. Please estimate the manure treatment cost per cubic meter of pig manure and wastewater, including collection, treatment, transportation, land application, labor, equipment maintenance, water, and electricity.

________ RMB/m^3

Q59. Does your farm have supporting cropland available for manure utilization?

○ Yes (please specify the area: ________ mu)

○ No

Q60. How are biogas slurry and biogas residue utilized? *(Multiple-choice)*

If more than one option is selected, please indicate the percentage allocated to each option. The percentages should sum to 100%.

□ Self-use (percentage: ________%)

□ Provided free of charge to others (percentage: ________%)

□ Sold (percentage: ________%)

Q61. If biogas slurry or residue is provided free of charge, how is it handled?

○ Direct cooperation with crop farmers

○ Processed through a third-party organization

Q62. If biogas slurry or residue is sold, how is it handled?

○ Direct cooperation with crop farmers

○ Processed through a third-party organization

Q63. Are there any costs associated with providing biogas slurry or residue free of charge (e.g., transportation, labor, and fuel)?

○ Yes (please specify annual expenditure: ________ RMB)

○ No

Q64. Are there any costs or revenues associated with selling biogas slurry or residue? *(Multiple-choice）*

□ Yes, costs incurred (please specify annual expenditure: ________ RMB)

□ Yes, revenue generated (please specify annual sales revenue: ________ RMB)

□ No costs and no revenues

Q65. Did your farm receive any government subsidies related to manure management? *(Multiple-choice）*

□ Subsidies for manure treatment facilities (e.g., urine storage tanks, composting sites, and other treatment or storage facilities) (amount: ________ 10,000 RMB)

□ Subsidies for manure treatment equipment (e.g., compost turners, solid-liquid separators, manure scrapers, and other manure treatment equipment) (amount: ________ 10,000 RMB)

□ Subsidies for manure treatment operations (amount: ________ 10,000 RMB)

□ No
