# Supplementary material for: Moderate Scale of Pig Farming: Implications for Herd Management and Disease Control—Evidence from Heilongjiang Province, China
Source: Vet Sci. 2026 Jul 24;13(8):728. doi: 10.3390/vetsci13080728 (PMC13517646; doi:10.3390/vetsci13080728)
Supplement: Supplementary file 1 [file vetsci-13-00728-s001.zip › Supplementary Material File S2.pdf]

Supplementary Tables. Definitions and descriptive statistics of variables used in the empirical analysis. Categorical variables are reported as counts and percentages of pig farms in each category, n (%), in Table S1. Continuous variables are reported as means and standard deviations (SD) in Table S2.

Table S1, Categorical variables

Variable category	Variable code	Variable name	Category	n (%)
Operating scale	S1	Annual slaughter volume	1–499 head	435 (53.8%)
	S2		500–1,499 head	177 (21.9%)
	S3		1,500–2,999 head	78 (9.6%)
	S4		3,000–4,999 head	75 (9.3%)
	S5		5,000–10,000 head	44 (5.4%)
Owner characteristics	DPT	Disease prevention training	No	491 (60.7%)
			Yes	318 (39.3%)
	GS	Government services	No	116 (14.3%)
			Yes	693 (85.7%)
	IBS	Internet-based services	No	713 (88.1%)
			Yes	96 (11.9%)
	FST	Farming skills training	No	369 (45.6%)
			Yes	440 (54.4%)
	MIS	Market information services	No	416 (51.4%)
			Yes	393 (48.6%)
Farm characteristics	FT	Farm topography	Plain/basin	137 (16.9%)
			Hilly area	295 (36.5%)
			Mountainous area	377 (46.6%)
	SITE	Farm site type	Leased	37 (4.6%)
			Self-built	772 (95.4%)
	LOAN	Farming loans	No	396 (49.0%)
			Yes	413 (51.1%)
Farm characteristics	INS	Pig insurance	No	257 (31.8%)
			Yes	552 (68.2%)

			Rarely	47 (5.8%)
			Once a month	204 (25.2%)
	CDF	Cleaning and disinfection frequency	Once a week	401 (49.6%)
			Two to three times a week	95(11.7%)
			Every day	62 (7.7%)
			1 point	0 (0%)
			2 points	16 (2.0%)
	BSL	Biosecurity level	3 points	230 (28.4%)
			4 points	303(37.5%)
			5 points	260 (32.1%)
			Water-flushed manure	152 (18.8%)
	MCM	Manure collection method	Water-soaked manure	201 (24.9%)
			Dry manure cleaning	456 (56.4%)
	SC	Supporting cropland	No	254 (31.4%)
			Yes	555 (68.6%)
	SBS	Self-use of biogas slurry	No	205 (25.3%)
			Yes	604 (74.7%)
	GBS	Giving away biogas slurry	No	348 (43.0%)
			Yes	461 (57.0%)
	SALBS	Sale of biogas slurry	No	766 (94.7%)
			Yes	43 (5.3%)
	MTS	Manure treatment subsidy	No	516 (63.8%)
			Yes	293 (36.2%)
			Frequent occurrence	36 (4.5%)
			Occasional occurrence	168 (20.9%)
External environmental characteristics	SDR	Surrounding disease risk	Rare occurrence	207 (25.6%)
			Basically no occurrence	289 (35.7%)
			Never occurred	108 (13.4%)

Table S2, Continuous variables

Variable category	Variable code	Variable name	Variable definition	Mean	SD
Input–output variables	OVPP	Output value per pig	Slaughter weight × selling price per kilogram (CNY/head)	2130.41	276.91
	FC	Feed cost	Unit feed cost (CNY/kg)	10.13	1.12
	PC	Piglet cost	Unit piglet cost (CNY/kg)	2.30	0.61
	LC	Labor cost	Unit labor cost (CNY/kg)	1.56	0.77
	MDPC	Medical and disease prevention cost	Unit medical and disease prevention cost (CNY/kg)	0.66	0.59
	OC	Other costs	Land cost, utilities, depreciation, etc. (CNY/kg)	1.06	0.55
Disease prevention and control performance	PMR	Pig mortality rate	Pig deaths/(slaughtered pigs + pig deaths) (%)	1.51	3.10
Environmental management cost	MTC	Manure treatment cost	Treatment cost per cubic meter of pig manure and urine (CNY/m^3)	14.26	3.25
Owner characteristics	AGE	Age	Age of the farm owner (years)	48.37	8.00
	EXP	Years of farming experience	Years engaged in pig farming	11.45	6.34

	EDU	Education level	Years of education of the farm owner	10.57	2.59
	FL	Farm labor	Number of hired laborers engaged in farming	1.35	1.88
Farm characteristics	DPI	Disease prevention investment	Investment in disease prevention facilities (10,000 CNY)	9.39	9.87
	TFI	Treatment facility investment	Total investment in manure treatment facilities (10,000 CNY)	28.88	46.22
